# Art and Cultural Participation and Life Satisfaction in Adults: The Role of Physical Health, Mental Health, and Interpersonal Relationships

**DOI:** 10.3389/fpubh.2020.582342

**Published:** 2021-01-21

**Authors:** Chia-Wen Lee, Li-Ching Lin, Huang-Chia Hung

**Affiliations:** ^1^College of Modern Management, Yango University, Fuzhou, China; ^2^Department of Art Industry, National Taitung University, Taitung, Taiwan; ^3^Department of Physical Education, National Taitung University, Taitung, Taiwan

**Keywords:** art and cultural participation, life satisfaction, interpersonal relationship, mental health, physical health, museums and art galleries

## Abstract

Many different forms of art and cultural participation, for example, visiting museums and galleries, have received increasing attention as an important new focus for public health. While a growing body of evidence suggests that art and cultural participation enhance human health and well-being in the West, the research is just in the infancy in the East. The purpose of this study was to explore the effects of art and cultural participation of museums and galleries on life satisfaction intervened and controlled by physical and mental health and interpersonal relationships and individual's background. This study adopted large-sample data from the seventh period of the Taiwan Social Change Survey. The sample population comprised 2,034 adult participants, involving 1,042 males and 992 females. The average age of the respondents was 47.67 ± 17.48 years. The results showed that individuals aged 30–64 years with an average monthly income between NT$20,000–NT$40,000 had a higher frequency of art and cultural participation of museums and galleries. Women under 29 years reported better life satisfaction. Individuals with a higher frequency of art and cultural participation of museums and galleries affected life satisfaction not only directly but also indirectly through interpersonal relationships, particularly among women and the elderly. Individuals who visited museums and galleries more frequently had greater life satisfaction and higher frequency of interpersonal contact. This was particularly evident in older females. Art and cultural participation of museums and galleries directly affected individual's life satisfaction and indirectly affected it via interpersonal relationships after controlling for gender, age, and monthly income. These findings indicate that visiting museums and galleries can enhance the frequency of interpersonal interactions and life satisfaction.

## Introduction

Art and cultural events are leisure activities that improve quality of life (1, 2). They offer physical, mental, social, educational, and aesthetic benefits, in addition to relaxation, resulting in improved life satisfaction (3, 4). In the Cultural White Paper issued by the Cultural Construction Committee of the Executive Yuan in Taiwan, it is stated that “culture is the foundation of the country, and the track of an individual's life depends on the accumulation of his or her behavioral and ideological growth.” The ultimate goal of cultural development is to enrich an individual's life satisfaction and quality of life, both of which are closely related to a country's development (5). Culture is the lifeline of a nation, and cultural growth is the driving force behind social progress (6). For these reasons, to enrich people's spiritual life and to promote the flourishing of culture, it is important to look at the topic of art and cultural participation from various perspectives.

Art and cultural participation such as visiting museums and art galleries has received increasing attention by many scholars (7–10). Especially, previous studies have verified that the participation can enhance human health and well-being (11, 12). To participate in museums and art galleries activities can increase the benefit of physical functioning for patients in hospital and care home (13–18). Chatterjee and Noble (19) point out that to engaging with museums and art galleries can lead to reduced anxiety and raised positive emotions, decreased social isolation, increased communications among families, and positive social experience (7). Visiting museums and art galleries is widely accepted as an essential index of art and cultural participation to provide the references of health and well-being for populations and local communities when analyzed and valued in a multidimensional, multi-attribute, and multi-value socioeconomic environment (20). Although there are robust studies regarding the efficacy of art and cultural participation in the West, the study in the East is still limited.

Studies have used objective and explicit indicators such as gender, ethnicity, educational qualification, socioeconomic status, and other background variables to explain life satisfaction (21–24). However, much research is inconsistent or contradictory owing to differences in the sample populations and control variables (25–27). Furthermore, the absence of intermediary variables between the applicable variables confuses the causal relationships between background variables with art, cultural participation, and life satisfaction (25). In recent years, researchers have explored how art and cultural participation promotes life satisfaction using multivariate statistical methods involving critical intermediate variables to analyze causal mechanisms (28–30). To clearly understand the relationship between art and cultural participation and life satisfaction, the following intermediating variables have been explored: sense of achievement (26), physical and mental health (31–34), interpersonal attachment and self-efficacy (30), and interpersonal relationships (22, 24, 27).

Physical and mental health factors are important variables affecting an individual's life satisfaction. Good physical health not only increases participation in various activities but also improves life satisfaction (33, 35). Moreover, good mental health improves attitudes and allows people to fulfill their desires, which in turn promotes life satisfaction. Notably, the quality of interpersonal relationships is another variable that influences life satisfaction: good interpersonal relationships and attachments and engaging in the community are key factors for improving life satisfaction (24, 25, 31). For these reasons, studies should explore the relationships between variables by addressing the factors that affect life satisfaction from the viewpoint of physical and mental health and interpersonal relationships.

Although previous studies supported that art and cultural participation was associated with individual's health and life satisfaction (26, 29, 30, 36), the factors of physical and mental health and interpersonal relationships and individual background had not been included as mediator and control variables, which are important in improving our understanding of the impact of art and cultural participation on individuals' life satisfaction. The purpose of this study was to investigate the influences of art and cultural participation of museums and galleries on life satisfaction intervened and controlled by physical and mental health and interpersonal relationships and background. Specifically, based on the large sample data from the basic survey database of social change in Taiwan and after controlling background variables, we expanded the survey samples and regions to explore the relationship between variables and conducted further research on the consideration of relevant units by using various statistical methods. Moreover, government agencies and nongovernmental organizations can take our findings as solid evidence to develop effective strategies to promote art and cultural participation in individuals to increase life satisfaction.

## Background Conception

### Art and Cultural Participation

Art and cultural activities entail leisure and happiness and have cognitive, emotional, spiritual, and educational functions that can help humans satisfy their need for knowledge (37). Beauty and self-realization can further enhance the public's appreciation of art, culture, and beauty (24). Culture and art experienced during adulthood are manifested through food habits, clothing, housing, education, and music. These societal elements constantly evolve with the pulse of life and are subsequently infused with new connotations (6). Along with social changes, art and culture present various facets of adulthood. All events with cultural significance can be defined as cultural activities. According to this definition, everything can be viewed as art or cultural activity. In a narrower sense, engagement with music, dance, art, drama, literature, on-site creative technology, or other domains of art and culture, recognized by competent authorities, can be considered art and cultural participation (37). Such pursuits satisfy human's thirst for knowledge, aesthetics, and self-realization. They can also improve one's ability to appreciate art, culture, and aesthetics. Additionally, participation refers to a person's involvement in an action or an event (38), characterized by physical and mental investment and interpersonal interaction behavior (11). In the current paper, because of the nature of the data collected through the Taiwan Social Change Survey, we focus on one specific aspect of art and cultural participation: visiting museums and galleries.

### Evaluation of Life Satisfaction

In academia, happiness, mental health, quality of life, and life satisfaction are the main indicators of individual living conditions (39, 40). Subjective well-being and life satisfaction are important determining factors (41). Happiness, mental well-being, quality of life, and life satisfaction have similar meanings; typically, they signify a positive mental state. These words are even used synonymously (42). Life satisfaction plays an important role in defining and evaluating the quality of life (43). Satisfaction is judged by comparing one's current situation with their ideal situation, the standards of which are set by individuals themselves rather than by others. Life satisfaction is defined as an individual's cognizance and evaluation of his own life and a comprehensive or specific evaluation of contributing factors (42). In short, life satisfaction is an individual's subjective cognizance and evaluation of his personal life situation based on appropriate standards.

Life satisfaction reflects an individual's conscious experience of inner pleasure and encourages the individual to actively pursue his or her goals (44). Individuals with specific goals are more capable of improving their life satisfaction because they can organize and integrate their resources to move toward their goals (45). Life satisfaction drives an individual to actively pursue a meaningful life. Many methods have been developed for assessing life satisfaction. For example, the Satisfaction With Life Scale (SWLS) utilizes the perspective of perceived self-life state (41). Comprehensive Quality of Life (ComQol) is evaluated through the perspective of material well-being, health, intimacy, safety, community, and emotional well-being (46). There remain several measurements, including the desire to improve life (47), personal satisfaction with the past and the future, self-assessment of personal life, the comparison of personal desire and actual achievement (48), the discussion of the quality of life and other mental and physical health indicators (49), the aspects of work and family fields, and personality traits (50). As mentioned above, research on life satisfaction should evaluate the impact of physiological, psychological, and social benefits on individual lifestyles.

### Art and Cultural Participation and Life Satisfaction

Art and cultural participation not only is an essential component of public leisure participation behavior but also effectively promotes individual life satisfaction (2). Studies have reported a positive correlation between leisure participation and life satisfaction (34). In particular, increased participation in art and cultural activities is related to increased life satisfaction (26). Hence, studying art and cultural participation can improve our understanding of life satisfaction.

Leisure participation provides physical, mental, and social benefits and in turn promotes life satisfaction (51, 52). Art and cultural events are considered leisure activities. The physiological and psychological experiences, satisfaction, and interpersonal relationship involved in the process of participation promote physiological, psychological, and social benefits (2, 53).

Physical and mental benefits and interpersonal relationships are related to life satisfaction. First, physical health has a positive effect on perceived life satisfaction. Compared with static leisure, participation in dynamic leisure activities can have additional benefits on life satisfaction as it improves physical health (7, 33, 35). Therefore, there is a strong association between individual physical health and life satisfaction. Secondly, while experiencing happiness, individuals feel satisfied and happy with their personal life. The more individuals engage in positive behavior, the less likely they are to experience negative emotions (41). Individuals can psychologically benefit from leisure participation, which are associated with numerous empirical outcomes (3). Moreover, leisure participation has a positive correlation with psychological well-being and shares a causal relationship with mental health and life satisfaction (54–56). Therefore, through leisure participation, individuals can achieve happiness, good mental health, and life satisfaction with positive benefits. Finally, social networks created through leisure participation are important for promoting life satisfaction. Many researchers believe that leisure participation at the social level can effectively improve individual happiness, relaxation, health, psychological state, happiness, and life satisfaction. Having good social relationships can promote interpersonal relations and consequently improve life satisfaction (2, 52, 53). Support from social networks and interpersonal relationships improves well-being (57, 58). Interpersonal variables may be a crucial factor affecting life satisfaction mediated by the similarities between well-being and life satisfaction. In short, interpersonal relationships are positively associated with life satisfaction.

## Methods

In this study, background variables such as gender, age, and average monthly income were controlled for. Physical and mental health and interpersonal relationships were used as intermediary variables to explore the influence of art and cultural participation on life satisfaction.

### Participants

We obtained data from the first comprehensive questionnaire of the seventh period of the Taiwan Social Change Survey (QTSCS) in 2015 (59). The sample population comprised 2,034 participants in Taiwan from various counties and cities, all older than 18 years. The study population included 1,042 males (51.2%) and 992 females (48.8%). The basic survey of social change in Taiwan is a long-term research project involving a sampling survey sponsored by the Humanities Department of the Ministry of Science and Technology. This research uses data collected since 1984 from a public database that has been extensively used for academic research. By the end of 2017, more than 1,900 studies have been conducted using data collected from this database (60).

### Measurements

#### Background

1. Gender: In the regression analysis, males were assigned a value of 1 and females 0.
2. Age: Year of birth.
3. Income: The monthly income of participants was recorded. The options were no income, <NT$10,000, <NT$20,000, and NT$30,000 or more, scored as 1, 2, or 3. Higher scores denoted higher average monthly incomes.

#### Art and Cultural Participation

The participants were asked about the number of times they had visited art and history museums and art galleries in the previous year. Points from 1 to 6 corresponded to no visit, once, twice, three times, four times, and more than five times, respectively.

#### Physical Health

Participants were asked if they experienced headaches, palpitations (rapid heartbeats), and worries about possible heart disease, tightness in the chest, extreme discomfort, shivering, or numbness (59). Points from 4 to 1 corresponded to never, sometimes, more than usual, and frequently, respectively. A higher score represents better physical health.

#### Mental Health

The participants were asked whether they felt burdened, lost, hopeless, nervous, or incapable of relaxing (59). Points from 4 to 1 corresponded to never, sometimes, more than usual, and frequently, respectively.

#### Interpersonal Relationship

The participants were asked how often they had visited their relatives and friends. Answers were recorded as never, very few times, once in 2 or 3 months, one to three times a month, once a week, and twice a week or more (59). The participants were evaluated with respect to the number of individuals that they communicated with within a single day, from morning to night. The answers were 0–4, 5–9, 10–19, 20–49, 50–99, and more than 100, and the responses were assigned 1, 2, 3, 4, 5, and 6 points, respectively.

#### Life Satisfaction

The participants were asked if they were satisfied with their current quality of life and their relationships with their friends (59). Scores from 4 to 1 corresponded to very satisfied, fairly satisfied, not satisfied, and very dissatisfied, respectively. Furthermore, participants were asked if they were happy with their current life, and scores from 4 to 1 corresponded to very happy, fairly happy, not very happy, and very unhappy, respectively.

### Data Analysis

Descriptive statistics, including the number of participants, means, standard deviation, and percentage values, were used to analyze the sample distribution. The survey data were subjected to exploratory factor analysis to identify the dimensions of QTSCS. Through the background variables, we analyzed the variance in art and cultural participation, physical and mental health, interpersonal relationships, and life satisfaction. If the variances were found to be significant, study comparisons were performed using the Scheffé method. The background variables were controlled for multiple regression analyses to investigate the effects of the following variables on life satisfaction: participation in art and cultural events; physical and mental health; and interpersonal relationships.

## Results

### Demographics

The sample population comprised 2,034 respondents, including 1,042 (51.20%) males and 992 (48.80%) females. The average age of the respondents was 47.67 ± 17.48 years. The average monthly income of 309 (20.16%), 273 (17.81%), 178 (11.61%), and 167 (10.89%) respondents were <NT$20,000–NT$30,000, NT$30,000–NT$40,000, NT$10,000–NT$20,000, and NT$40,000–NT$50,000, respectively. The remaining respondents comprised 10% of the population.

### Factor Analysis

The objective of factor analysis was to explore the grouping between the 11 questionnaires. The study was to utilize factors with a latent root or eigenvalue > 1 (61). Items with factor loading of 0.4 or higher were considered acceptable variables to measure constructs (62). This approach produced four factors for the data, and this is supported by a scree test (61). Based on an assessment of the factor loading scores, the following four dimensions were proposed: (1) Physical health (eigenvalue = 2.44, explained variance = 17.39%), (2) mental health (eigenvalue = 2.35, explained variance = 16.79%), (3) life satisfaction (eigenvalue = 1.77, explained variance = 12.62%), (4) interpersonal relationship (eigenvalue = 1.41, explained variance = 10.06%). The four with eigenvalues that exceeded 1 were extracted, and it was found that these explained 56.86% of the overall variance in the data (Table 1). (63) suggested that over 50% of the overall variance was reasonable for a data set of this kind. Therefore, four factors of physical health, mental health, and interpersonal relationship were identified as the main factors.

**Table 1:** Exploratory factor analysis of QTSCS.

Dimensions of QTSCS^*^	**Physical health**	**Mental health**	**Interpersonal relationship**	**Life satisfaction**
Feel headache or tight head?	0.64			
Feel palpitation or palpitation, worry about possible heart disease?	0.77			
Feel tight chest, very uncomfortable?	0.80			
Feel your hands and feet shaking or numb?	0.65			
Feel that many things are a burden to you?		0.66		
Feel lost confidence yourself?		0.84		
Feel hopeless in life?		0.78		
Feel nervous and can't relax?		0.72		
Do you and your relatives get together frequently?			0.66	
Do you and your friends often get together?			0.70	
How many people do you contact from morning to night in a day?			0.64	
Are you satisfied with your current quality of life?				0.74
Are you satisfied with your relationship with your friends?				0.75
Do you feel happy at present?				0.72
Eigenvalue	2.44	2.35	1.41	1.77
% of variance explained (Total = 56.86%)	17.39%	16.79%	10.06%	12.62%

* *Questionnaire of the seventh period of the Taiwan Social Change Survey*.

### Factors Influencing Life Satisfaction

The regression analysis of life satisfaction (1), background variable, revealed that female gender (β = −0.10, *p* = 0.00 < 0.05) and monthly income (β = 0.09, *p* = 0.00 < 0.05) had significantly positive effects on life satisfaction (Table 1). Similarly, regression analysis of life satisfaction (2), background variable, revealed that female gender (β = −0.09, *p* = 0.00 < 0.05), age (β = 0.07, *p* = 0.13 < 0.05), and art and cultural participation (β = 0.07, *p* = 0.01 < 0.05) had significantly positive effects on life satisfaction. Regression analysis of life satisfaction (3), background, physical health, mental health, and interpersonal relationship variables indicated that female gender (β = −0.11, p = 0.00 < 0.05), age (β = 0.10, *p* = 0.00 < 0.05), art and cultural participation (β = 0.05, *p* = 0.05 < 0.05), physical health (β = 0.06, *p* = 0.02 < 0.05), mental health (β = 0.07, *p* = 0.01 < 0.05), and interpersonal relationships (β = 0.16, *p* = 0.00 < 0.05) had significantly positive effects on life satisfaction. However, monthly income (β = 0.05, *p* = 0.10 > 0.05) was not a significant factor in life satisfaction, with an overall variance of 5.6% (Table 2). Additionally, the regression analysis suggests that a higher frequency of participation in art and cultural participation was positively associated with physical and mental health, interpersonal relationships, and life satisfaction, which was particularly evident in older women.

**Table 2:** Regression analysis of physical health, mental health, interpersonal relationship, and life satisfaction.

	PH^*^ (1)	PH^*^ (2)	MH^*^ (1)	MH^*^ (2)	IR^*^ (1)	IR^*^ (2)	LS^*^ (1)	LS^*^ (2)	LS^*^ (3)
**Variables**	**β**	**β**	**β**	**β**	**β**	**β**	**β**	**β**	**β**
Male (female as reference)	−0.09^**^	−0.09^**^	−0.02	−0.02	0.04	−0.05	−0.10^**^	−0.09^**^	−0.11^**^
Age	0.02	−0.02	0.11^**^	11^*^	−0.22^**^	−0.21^**^	0.06	0.07^**^	0.10^**^
Monthly income	−0.06^**^	−0.07^**^	−0.08^**^	−0.08^**^	0.16^**^	−0.13^**^	0.09^**^	0.07^**^	0.05
Art and cultural participation		0.03		−0.01		0.14^**^		0.07^**^	0.05^**^
PH									0.06^**^
MH									0.07^**^
IR									0.16^**^
R square	0.014	0.012	0.022	0.23	0.093	0.111	0.015	0.020	0.056
Adj. R square	0.014	0.012	0.021	0.20	0.091	0.108	0.013	0.017	0.051

* *PH, physical health; MH, mental health; IR, interpersonal relationship; LS, life satisfaction*.

** *p < 0.05*.

### Criterion Variables of Physical and Mental Health and Interpersonal Relationships

In the regression analysis, background variables were controlled to determine whether participation in art and cultural participation influenced life satisfaction through intermediary variables, such as physical health, mental health, and interpersonal relationships. These were considered criteria to determine whether the individual's background variables influenced life satisfaction through intermediary variables (Table 2).

Regression analysis of physical health (1), background variable, indicated that female gender (β = −0.09, *p* = 0.00 < 0.05) and monthly income (β = −0.06, *p* = 0.02 < 0.05) had significantly positive effects on physical health. Regression analysis of physical health (2), background variable, revealed that female gender (β = −0.09, *p* = 0.00 < 0.05) and monthly income (β = −0.07, *p* = 0.01 < 0.05) had significantly positive effects on physical health. However, art and cultural participation (β = 0.03, *p* = 0.28 > 0.05) was not significantly correlated with physical health, with an overall explained variance of 1.2%. This result suggests that art and cultural participation is not correlated with physical health.

Regression analysis of physical health (1), background variable, revealed that age (β = 0.11, *p* = 0.00 < 0.05) and monthly income (β = −0.08, *p* = 0.00 < 0.05) had significantly positive effects on physical health. The regression analysis of physical health (2), background variable, indicated that age (β = 0.11, *p* = 0.00 < 0.05) and monthly income (β = −0.08, *p* = 0.00 < 0.05) had significantly positive effects on physical health. However, female gender (β = −0.02, *p* = 0.92 > 0.05) and art and cultural participation (β = −0.01, *p* = 0.83 > 0.05) were not significantly associated with mental health, and the overall explained variance was 2.0%. These results suggest that art and cultural participation was not correlated with physical health.

Regression analysis of physical health (1), background variable, indicated that age (β = −0.22, *p* = 0.00 < 0.05) and monthly income (β = 0.16, *p* = 0.00 < 0.05) had a significantly positive effect on interpersonal relationships. Regression analysis of physical health (2), background variable, indicated that age (β = −0.21, *p* = 0.00 < 0.05), monthly income (β = 0.13, *p* = 0.00 < 0.05), and art and cultural participation (β = 0.140, *p* = 0.00 < 0.05) had a significantly beneficial effect on interpersonal relationships. However, female gender (β = −0.02, *p* = 0.10 > 0.05) was not significantly associated with interpersonal relationships, and the overall explained variance was 11.0%. These results demonstrated that art and cultural participation was not correlated with physical health.

### A Path Analysis of Life Satisfaction

**Figure 1:**
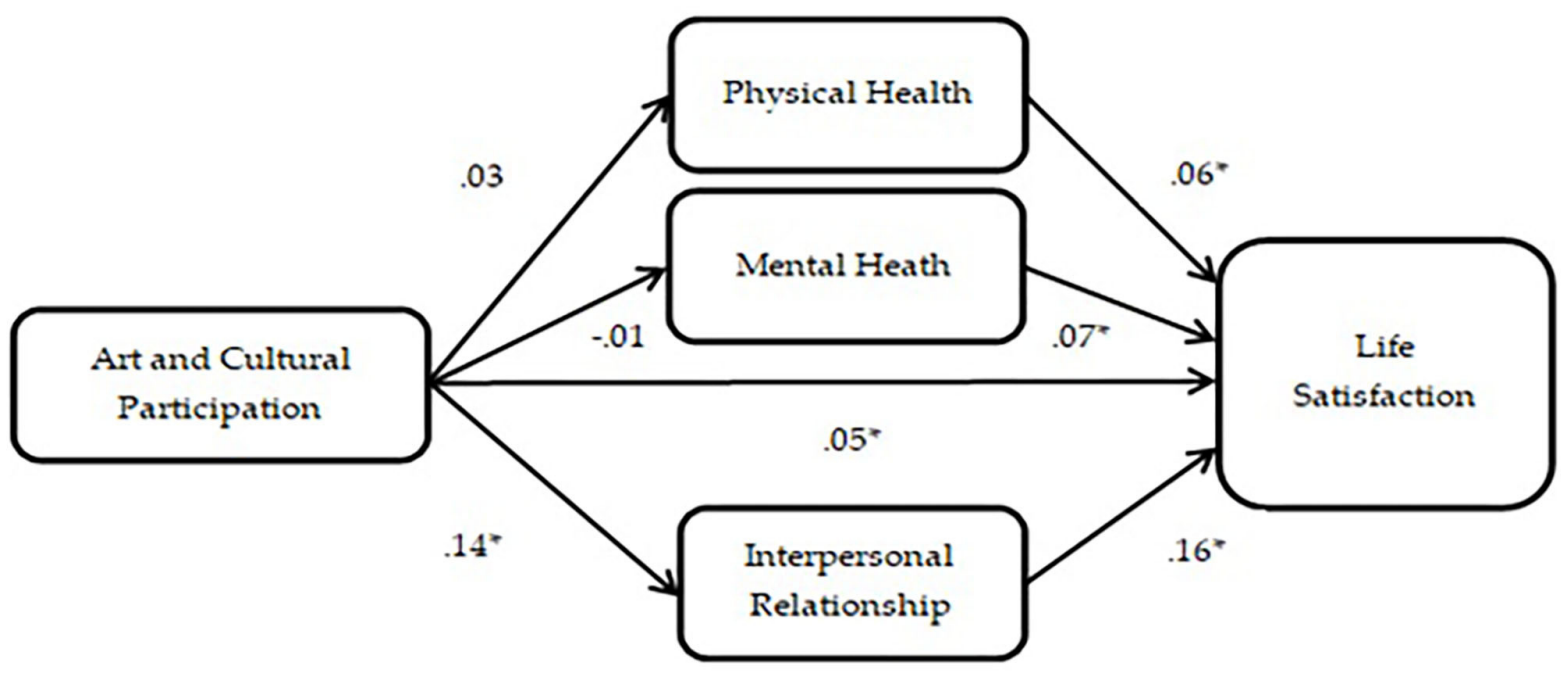
Path analysis of art and cultural participation on life satisfaction.

Figure 1 shows that arts and cultural participation had a positively direct effect (β = 0.5, *p* = 0.05 < 0.05) on life satisfaction and an indirect effect through the development of interpersonal relationships (β = 0.02, *p* = 0.00 < 0.05). Physical health (β = 0.06, *p* = 0.02 < 0.05) and mental health (β = 0.07, *p* = 0.01 < 0.05) only affected life satisfaction by controlling the background variables, gender, age, and monthly income. Art and cultural participation did not significantly influence physical health (β = 0.03, *p* = 0.28 > 0.05) or mental health (β = −0.01, *p* = 0.83 > 0.05).

## Discussion

After controlling for background variables (gender, age, and monthly income), individual art and cultural participation had direct effects on life satisfaction and indirect effects through the development of interpersonal relationships. Individuals aged 30–64 years with average monthly income of NT$20,000–NT$40,000 had more participation in art and cultural activities. Women under 29 years reported improved life satisfaction. A higher frequency of art and cultural participation not only directly affected life satisfaction but also indirectly affected satisfaction via the development of interpersonal relationships during these activities, particularly in women and the elderly. Moreover, good physical health and mental health were beneficial in developing positive personal attitudes toward life. Women's life satisfaction was higher than that of men, consistent with findings of previous studies. This could be attributed to women's traditional aesthetic training in Taiwan's social environment. Women establish their self-identity and improve their life satisfaction by cultivating their artistic taste (64). This finding is also consistent with the theory of cultural and financial capital (53). It is believed that older women spend more capital in art and cultural activities, in turn improving their life satisfaction.

Art and cultural participation enhances interpersonal relationships and mutual understanding. In this study, art and cultural activities had a positive impact on interpersonal relationships, which is consistent with findings of studies that suggested that art and cultural participation increases opportunities for interpersonal communication and relationships (Chatterjee and Noble, 2013) (21, 51). The weak state of interpersonal relationships in modern society demonstrates the value of art and cultural participation. Notably, individuals with increased art and cultural participation reported greater levels of life satisfaction, which was consistent with findings of previous studies (2). Specific cultural resources, such as art and cultural performances, cultural publishing, film and television, music, and cultural exchanges and consumption, improved individual life satisfaction. Some studies have also indicated that the frequency of individual's art and cultural participation had a positive relationship with his or her life satisfaction (65). Art and cultural participation has been proposed to relax the body and mind, improve self-development, enhance life enjoyment and social integration, and promote health (66), all of which positively affects individual satisfaction with conditions of life. A recent study suggested that individuals with good taste in art and culture, self-worth, and self-dignity demonstrate multifaceted life satisfaction (26). These studies contribute to the existing research and establish that art and cultural participation can promote social interactions (67). In particular, individual participation in art and culture can promote good interpersonal relationships and life satisfaction.

The results suggest that physical health and mental health are determining factors that can contribute to life satisfaction, findings consistent with those of previous literature. A psychologically healthy individual is more capable of experiencing happiness, making it easier for him or her to achieve a positive evaluation of life, happiness, and satisfaction with life (29, 55, 56). Art and cultural activities have cognitive, emotional, spiritual, and educational functions that help humans meet their demands for knowledge, beauty, and self-realization. Encouraging individuals to appreciate art, culture, and aesthetics improves life satisfaction.

Interpersonal relationships have positive impacts on life satisfaction. In fact, studies have suggested that, if individuals get along with each other, their willingness to create social networks increases and in turn promotes their life satisfaction (2). Moreover, the quality of interpersonal interactions, interpersonal attachment, sense of community, and interpersonal relationships are factors that affect an individual's life satisfaction (25, 28). Taken together, these findings suggest that individuals with good interpersonal communication opportunities and interactions promote interpersonal relationships and enhance their life satisfaction.

## Conclusion and Suggestions

We found that increased participation in art and cultural participation affects an individual's life satisfaction not only directly but also indirectly via interpersonal relationships, and this was particularly evident for women and the elderly. Effective use of leisure time can offer personal, physical, and psychological benefits and can improve interpersonal relationships, in turn promoting life satisfaction (30, 53). We suggest that government agencies should evaluate measures to increase art and cultural events to promote life satisfaction in individuals. For example, providing more opportunities for teaching art and cultural events in school education would be an effective means of accomplishing this objective. Public and private organizations should provide information on art and cultural events through media platforms, encouraging individuals to participate in these events.

On the face of it, it appears plausible that art and cultural participation should have a significant impact on individual's quality of life or any other indicator of physical health and well-being (Chatterjee and Noble, 2013); however, this study did not find the relationship between them. There may also be the problem of reverse causality—people who are healthy and have good psychological well-being may be more likely to engage with cultural opportunities. In future research, we can explore the reverse causal relationship between art and culture participations and health from many aspects. People who are healthy and happy would tend to participate in art and cultural activities more.

After controlling for the variables of gender, age, and average monthly income, we found that high levels of participation in art and cultural activities and strong interpersonal relationships promote life satisfaction. Many other important variables can be considered to examine how art and cultural participation influences life satisfaction. For example, personality characteristics such as perception-seeking and experience-seeking can determine an individual's satisfaction with activities. Moreover, marital status and ethnic backgrounds affect participation in art and cultural activities in different ways. For example, married individuals and those with children have restrictions and obligations, limiting the types of activities they choose to participate in Qiu and Chen (68). Studies have shown that social support systems are important for personal, physical, and mental health, and they affect life satisfaction (69, 70). Future studies can include the variables of marriage and ethnic backgrounds, personality characteristics, and social support systems to fully understand the determining factors of life satisfaction.

This study used information from an existing database (QTSCS) for analysis. Although it employs a large sample size, with high efficiency, wide age range, and good representativeness, the analysis and interpretation of variables have limitations caused by the content planning of the questionnaire. For example, the questions concerning physical health variables are biased toward physiological cognition and less toward behavioral aspects, the latter of which warrants further investigation. Further research on the effect of physical and mental health and interpersonal relationships on life satisfaction is warranted. Placing questions into an appropriate category can clarify the relationships among these research variables.

## Data Availability Statement

Publicly available datasets were analyzed in this study. This data can be found here: https://srda.sinica.edu.tw/datasearch_detail.php?id=1057.

## Author Contributions

H-CH and L-CL contributed to the conceptualization, methodology, and data analysis. L-CL, C-WL, and H-CH contributed to writing the original draft preparation. C-WL and H-CH contributed to writing, review, and editing. All authors contributed to the article and approved the submitted version.

## Conflict of Interest

The authors declare that the research was conducted in the absence of any commercial or financial relationships that could be construed as a potential conflict of interest.

## References

[1] Kleiber DA Rickards WH Leisure and recreation in adolescence: limitation and potential. In: Wade MG, editor. Constraints on Leisure. Springfield, IL: Thomas (1985). p. 289–317.

[2] Kleiber DA Walker GJ Mannell RC A Social Psychology of Leisure. State College, PA: Venture Publishing, Inc (2011).

[3] Beard JG Ragheb MG Measuring leisure satisfaction. J Leisure Res. (1980) 12:20–33. 10.1080/00222216.1980.11969416

[4] Lu L Argyle M. Leisure satisfaction and happiness as a function of leisure activity. Kaohsiung J Medical Sci. (1994) 10:89–96. 8176776

[5] Ministry of Culture Culture White Book. (2020). Available online at: http://mocfile.moc.gov.tw/mochistory/images/policy/1998YellowBook/index.htm (assessed June 4, 2020).

[6] Council for Cultural Affairs, Executive Yuan 2004 Cultural White Book. Taiwan: Council for Cultural Affairs, Executive Yuan (2004).

[7] Chatterjee HJ Camic PM The health and wellbeing potential of museums and art galleries. Arts Health. (2015) 7:183–6. 10.1080/17533015.2015.1065594

[8] Clift S Camic PM Oxford Textbook of Creative Arts, Health and Wellbeing: International Perspectives on Practice, Policy and Research. Oxford, New York: Oxford University Press (2015).

[9] Cuypers K Krokstad S Lingaas Holmen TL Skjei Knudtsen M Bygren LO Holmen J. Patterns of receptive and creative cultural activities and their association with perceived health, anxiety, depression and satisfaction with life among adults: the HUNT study, Norway. J Epidemiol Commun Health. (2012) 66:698–703. 10.1136/jech.2010.113571 21609946

[10] O'Neil M Cultural attendance and public mental health: From research to practice. J Public Ment Health. (2010) 9:22–9. 10.5042/jpmh.2010.0700

[11] Camic PM Chatterjee HJ Museums and art galleries as partners for public health interventions. Perspect Public Health. (2013) 133:66–71. 10.1177/1757913912468523 23308010

[12] Napier AD Ancarno C Butler B Calabrese J Chater A Chatterjee H. Culture and health. Lancet. (2014) 384:1607–39. 10.1016/S0140-6736(14)61603-2 25443490

[13] Ander EE Thomson LJ Blair K Noble G Menon U Lanceley A Using museum objects to improve wellbeing in mental health service users and neurological rehabilitation clients. Br J Occup Ther. (2013) 76:208–16. 10.4276/030802213X13679275042645

[14] Ander E Thomson L Noble A Lanceley U Menon G Chatterjee HJ Heritage, health and wellbeing: assessing the impact of a heritage focused intervention on health and wellbeing. Int J Herit Stud. (2013) 19:229–42. 10.1080/13527258.2011.651740

[15] Lanceley A Noble G Johnson M Balogun N Chatterjee HJ Menon U. Investigating the therapeutic potential of a heritage-object focused intervention: a qualitative study. J Health Psychol. (2012) 17:809–20. 10.1177/1359105311426625 22104664

[16] Paddon H Thomson L Lanceley A Menon U Noble G Chatterjee HJ. Evidence of wellbeing benefits from a heritage-in-health intervention with hospital patients: a mixed methods approach. Arts Health. (2013) 6:24–58. 10.1080/17533015.2013.800987 25621005 PMC4285724

[17] Solway R Camic PM Thomson LJ Chatterjee HJ Material objects and psychological theory: a conceptual literature review. Arts Health. (2016) 8:82–101. 10.1080/17533015.2014.998010

[18] Thomson LJ Chatterjee HJ Measuring the impact of museum activities on wellbeing: developing the museum wellbeing measures toolkit. J Mus Manage Curatorship. (2015) 30:44–62. 10.1080/09647775.2015.1008390

[19] Chatterjee H Noble G Museums, Health and Well-Being, 1st ed. Routledge (2013). 10.4324/9781315596549

[20] Jensen A Stickley T Edgley A The perspectives of people who use mental health services engaging with arts and cultural activities. Ment Health Soc Inclus. (2016) 20:1–12. 10.1108/MHSI-02-2016-0011

[21] Freire T Teixeira A. The influence of leisure attitudes and leisure satisfaction on adolescent's posititve fucationing: the role of emotion regulation. Front Psychol. (2018) 9:1349. 10.3389/fpsyg.2018.01349 30123158 PMC6085571

[22] Matud MP García MC Fortes D. Relevance of gender and social support in self-rated health and life satisfaction in elderly Spanish people. Int J Environ Res Public Heal. (2019) 16:2725. 10.3390/ijerph16152725 31370147 PMC6695653

[23] Shin J Kim JK. How a good sleep predicts life satisfaction: The role of zero-sum beliefs about happiness. Front Psychol. (2018) 9:1589. 10.3389/fpsyg.2018.01589 30210411 PMC6121950

[24] Zou T Su Y Wang Y Examining relationships between social capital, emotion experience and life satisfaction for sustainable community. Sustain. (2018) 10:2651 10.3390/su10082651

[25] Chen WY Wang ML Wu CM The mediating influence of self-efficacy in the relationship attachment style and life satisfaction. Com Manage Quart. (2017) 18:101–21. Available online at: http://www.cabmt.org.tw/uploads/upload/ada8aaa7da9f947425516ac185a8eaca.pdf

[26] Lin LC Lee CW Hung HC Shih NM The Influence of people's cultural activities participation and sense of gain on life satisfaction. Leis Soc Res. (2018) 17:75–84. Available online at: http://lawdata.com.tw/tw/detail.aspx?no=336194

[27] Park S Sok SR. Relation modeling of factors influencing life satisfaction and adaptation of Korean older adults in long-term care facilities. Int J Environ Res Public Heal. (2020) 17:317. 10.3390/ijerph17010317 31906473 PMC6981623

[28] Chen YC Yang C Feng S. The effect of social communication on life satisfaction among the rural elderly: a moderated mediation model. Int J Environ Res Public Heal. (2020) 16:3791. 10.3390/ijerph16203791 31600931 PMC6843451

[29] Fu YC Lu L Chen SY Differentiating personal facilitators of leisure participation: socio-demographics, personality traits, and the need for sociality. J Tour and Leis Stud. (2009) 15:187–212. 10.6267/JTLS.2009.15(3)1

[30] Povedano-Diaz A Muñiz-Rivas M Vera-Perea M. Adolescents' life satisfaction: the role of classroom, family, self-concept and gender. Int J Environ Res Public Heal. (2020) 17:19. 10.3390/ijerph17010019 31861387 PMC6982278

[31] Chen SM Chang CC Wu CF Health improvement, interpersonal relationships, and life satisfaction among elderly learners in Taipei. J Res Edu Sci. (2018) 63:127–61. 10.6209/JORIES.201806_63(2).0006

[32] Lee SL Yen MK Leisure flow experience flow experience in leisure quality of life. Rev Leis Sport Health. (2011) 2:44–78. 10.29503/RLSH.201106.0004

[33] Gao JC Lin PQ Huang HM Lin YH The relationship between physical activity and quality of life in community-care-site older adults. J Nurse Healthcare Res. (2013) 9:157–67. 10.6225/JNHR.09.2.157

[34] Kim I Choi H Davis HT Health-related quality of life by the type of physical activity in Korea. J Commun Health Nurs. (2010) 27:96–106. 10.1080/07370011003704990 20437290

[35] Chang YZ Chen JH The study on the relationships among stress, leisure coping and quality of life for male HIV infection people. J Outdoor Recreat Stu. (2010) 23:51–77. 10.6130/JORS.2010.23(4)3

[36] Mak HW Coulter R Fancourt D. Does arts and cultural engagement vary geographically? Evidence from the UK household longitudinal study. Public Heal. (2020) 185:119–26. 10.1016/j.puhe.2020.04.029 32619767 PMC7456771

[37] Li YY A Study on Kaohsiung Citizens' Cultural Needs and Participation. KA, Taiwan: Research, Development and Evaluation Commission (1984).

[38] Wehmeier S Oxford Wordpower Dictionary. Oxford, NY: Oxford University Press (1993).

[39] Chen SY Fu YC Leisure participation and enjoyment among the elderly: individual characteristics and sociability. Educ Gerontol. (2008) 34:871–89. 10.1080/03601270802115382

[40] Fuligni AJ Hardway C Daily variation in adolescents' sleep, activities, and psychological well-being. J Res Adole. (2006) 16:353–78. 10.1111/j.1532-7795.2006.00498.x

[41] Lu L The meaning, measure, and correlates of happiness among Chinese people. Hum Soc Sci. (1998) 8:115–37.

[42] Diener E Emmons RA Larsen RJ Griffin S. The satisfaction with life scale. J Person Assess. (1985) 49:71–5. 10.1207/s15327752jpa4901_13 16367493

[43] Bailey TC Eng W Frisch MB Snyder CR Hope and optimism as related to life satisfaction. J Posit Psychol. (2007) 2:168–75. 10.1080/17439760701409546

[44] Frisch MB Clark MP Rouse SV Rudd MD Paweleck JK Greenstone A. Predictive and treatment validity of life satisfaction and the Quality of Life Inventory. Assess. (2005) 12:66–78. 10.1177/1073191104268006 15695744

[45] Emmons RA Personal strivings: an approach to personality and subjective well-being. J Personal Soc Psychol. (1986) 51:1058–68. 10.1037/0022-3514.51.5.1058

[46] Cummins RA The domains of life satisfaction: an attempt to order chaos. Soc Indicat Res. (1996) 38:303–28. 10.1007/BF00292050

[47] Cummins RA Mccabe MP Romeo Y Gullone E Validity studies the comprehensive quality of life scale (Comqol): instrument development and psychometric evaluation on college staff and students. Educ Psychol Measure. (1994) 54:372–82. 10.1177/0013164494054002011

[48] Diener E Suh EH Lucas RE Smith HL Subjective well-being: three decades of progress. Psychol Bull. (1991) 125:276–302. 10.1037/0033-2909.125.2.276

[49] Cribb K Life Satisfaction and Who Has It. (2000). Available online at: http://www.webclearinghouse.net/volume/3/-LifeSatisf.php (assessed June 4, 2020).

[50] Prasoon R Chaturvedi KR Life satisfaction: a literature review. Intern J Manage Humanit Soc Sci. (2016) 1:25–32. Available online at: https://pdf4pro.com/amp/cdn/life-satisfaction-a-literature-review-the-researcher-33518.pdf

[51] Driver BL Brown PJ Peterson GL Research on leisure benefits: an introduction to this volume. In: Driver BL Brown PJ Peterson GL, editors. Benefits of Leisure. State College, PA: Venture Publishing, Inc (1992). p. 3–12.

[52] Tinsley HEA Tinsley DJ A theory of attributes, benefits, and causes of leisure experience. Leis Sci. (1986) 8:1–45. 10.1080/01490408609513056

[53] Kelly JR Godbey G The Sociology of Leisure. State College, PA: Venture Publishing, Inc. (1992).

[54] Haworth JT Hill S Work, leisure, and psychological well-being in a sample of young adults. J Commun Appl Soc Psychol. (1992) 2:147–60. 10.1002/casp.2450020210

[55] Haworth J Lewis S Work, leisure and well-being. Br J Guid Counsel. (2005) 33:67–79. 10.1080/03069880412331335902

[56] Smale BJA Iwasaki Y Longitudinal analyses of the relationships among life transitions, chronic health problems, leisure, and psychological well-being. Leis Sci. (1998) 20:25–52. 10.1080/01490409809512263

[57] Cohen S Wills TA. Stress, social support, and the buffering hypothesis. Psycol Bull. (1985) 98:310–57. 10.1037/0033-2909.98.2.310 3901065

[58] House JS Umberson D Landis KR Structure and Social Indicat. Res. Ann Rev Sociol. (1998) 14:293–318. 10.1146/annurev.so.14.080188.001453

[59] Fu YC 2015 Taiwan Social Change Survey (Round 7, Year 1): Globalization, Work, Family, Mental Health, Religion, Mass Communication, Political Participation, Leisure. (2016). Available online at: https://srda.sinica.edu.tw/datasearch_detail.php?id=1057 (assessed August 31, 2020).

[60] Fu YC Introduction of 2018 Taiwan Social Change Survey. (2018). Available online at: https://www.most.gov.tw/most/attachments/9e154fde-8e5b-422b-a2d0-5ef4dfba2a53 (assessed December 4, 2020).

[61] Hair J Anderson R Tatham R Black W Multivariate Data Analysis. 5th ed Upper Saddle River, NJ: Prentice Hall (1998).

[62] Tinsley HE Kass RA The latent structure of the need satisfying properties of leisure activities. J Leis Res. (1979) 11:278 10.1080/00222216.1979.11969406

[63] Cohen J Statistical Power Analysis for the Behavioral Science. 2nd ed Hillsdale, NJ: Lawrence Erlbaum Associate (1988).

[64] Chang WC Kuo LM The relationship between identity and life satisfaction- a study on the taste for art. J Taiwan Stud. (2008) 5:1–41. 10.6456/JTS.200812.0001

[65] Sjögren K Hansson EE Stjernberg L. Parenthood and factors that influence outdoor recreational physical activity from a gender perspective. BMC Pub Heal. (2011) 11:2–9. 10.1186/1471-2458-11-93 21310038 PMC3045950

[66] Lu L Hu CH Experiencing leisure: the case of Chinese university students. Fu Jen Stud Sci Eng. (2002) 36:1–21.

[67] Fu YC Better together? Sociability in leisure and leisure satisfaction. Taiwa. J Sociol. (2009). 42, 55–94. Available online at: https://www.ios.sinica.edu.tw/people/personal/fuyc/%E7%8D%A8%E6%A8%82%E6%A8%82%E4%B8%8D%E5%A6%82%E7%9C%BE%E6%A8%82%E6%A8%82.pdf

[68] Qiu JW Chen ZC A Study on Folk Art And Cultural Activities And Consumer Behavior in Changhua County. Changhua, Taiwan: Changhua County Cultural Affairs Bureau (2011).

[69] Jou YH Chuang YL The transformation of stressors in late life, social supports, and the metal and physical health of the elderly: a longitudinal study. J Soc Sci Phil. (2000) 12:277–265.

[70] Ross CE Wu CL. The links between education and health. Am Sociol Rev. (1995) 60:719–45. 10.2307/209631928991080

